# Folate-Equipped Nanolipoplexes Mediated Efficient Gene Transfer into Human Epithelial Cells

**DOI:** 10.3390/ijms14011477

**Published:** 2013-01-14

**Authors:** Emmanuel Mornet, Nathalie Carmoy, Céline Lainé, Loïc Lemiègre, Tony Le Gall, Isabelle Laurent, Remi Marianowski, Claude Férec, Pierre Lehn, Thierry Benvegnu, Tristan Montier

**Affiliations:** 1 INSERM U1078, IFR 148 ScInBIoS, Université de Bretagne Occidentale, 46 rue Félix Le Dantec, CS 51819, 29218 Brest Cedex 2, France; E-Mails: emmanuel.mornet@chu-brest.fr (E.M.); nathalie.carmoy@univ-brest.fr (N.C.); tony.legall@univ-brest.fr (T.L.G.); claude.ferec@univ-brest.fr (C.F.); pierre.lehn@univ-brest.fr (P.L.); 2 Ecole Nationale Supérieure de Chimie de Rennes, CNRS, UMR 6226, Avenue du Général Leclerc, CS 50837, 35708 Rennes Cedex 7, France; E-Mails: celine.laine@free.fr (C.L.); loic.lemiegre@ensc-rennes.fr (L.L.); isabelle.laurent@u-cergy.fr (I.L.); thierry.benvegnu@ensc-rennes.fr (T.B.); 3 Service d’ORL et de chirurgie cervico-faciale, CHU de BREST hôpital Morvan, 2 avenue du maréchal Foch 29609 Brest, France; E-Mail: Remi.Marianowski@chu-brest.fr; 4 IBiSA “SynNanoVect” platform, IFR 148 ScInBIoS, Faculté de Médecine, Université de Bretagne Occidentale, 22 avenue Camille Desmoulins, CS 93837–29238 Brest cedex, France; 5 DUMG – Faculté de médecine, Université de Bretagne Occidentale, 22 avenue Camille Desmoulins, CS 93837–29238 Brest cedex, France; 6 CHRU de Brest, hôpital Morvan, 2 avenue du maréchal Foch 29609 Brest, France

**Keywords:** cystic fibrosis, cationic lipids, neutral lipid nanocomplexes, folic acid, folate receptors, epithelial cells

## Abstract

Since recombinant viral vectors have been associated with serious side effects, such as immunogenicity and oncogenicity, synthetic delivery systems represent a realistic alternative for achieving efficacy in gene therapy. A major challenge for non-viral nanocarriers is the optimization of transgene expression in the targeted cells. This goal can be achieved by fine-tuning the chemical carriers and the adding specific motifs to promote cellular penetration. Our study focuses on the development of novel folate-based complexes that contain varying quantities of folate motifs. After controlling for their physical properties, neutral folate-modified lipid formulations were compared *in vitro* to lipoplexes leading to comparable expression levels. In addition, no cytotoxicity was detected, unlike what was observed in the cationic controls. Mechanistically, the delivery of the transgene appeared to be, in part, due to endocytosis mediated by folate receptor targeting. This mechanism was further validated by the observation that adding free folate into the medium decreased luciferase expression by 50%. *In vivo* transfection with the folate-modified MM18 lipid, containing the highest amount of FA-PEG_570_-diether co-lipid (*w:w*; 90:10), at a neutral charge ratio, gave luciferase transgene expression. These studies indicate that modification of lipids with folate residues could enhance non-toxic, cell-specific gene delivery.

## 1. Introduction

Many diseases caused by genetic anomalies (e.g., mutation, aberrant expression) are currently not treatable by conventional therapies. Recent scientific advances in genomics have enhanced our understanding of the role that genes play in disease and have led to gene therapy, one of the most promising biomedical and pharmacological interventions, which has already seen clinical success [1]. Gene therapy relies on the introduction of functional nucleic acid sequences into target cells to treat the pathology associated with diseases, such as in cystic fibrosis (CF) [2,3]. In addition to inherited disorders like CF [4], gene therapy could potentially be used as a standard clinical intervention for the treatment of cancer, infectious diseases [5], cardiovascular disorders [6], and neurological pathologies [7], to name but a few. Since the approval of the first gene therapy clinical trial in the USA in early 1990’s [8], gene therapy has evolved rapidly throughout the world and has been the basis for a plethora of biomedical and biotechnology companies. To date, more than 1400 gene therapy clinical trials have been completed, are ongoing, or have been approved worldwide [9].

Although viruses are the most effective means to deliver DNA, their effectiveness has been overshadowed by immune issues, transgene random integration and viral recombination; all of which could potentially compromise patient safety [10,11]. As an alternative to the viruses, synthetic reagents offer features that circumvent the issues limiting virus-based clinical trials. However, the relatively low transfection efficiency associated with synthetic reagents needs to be addressed before these delivery systems can be used for clinical benefit.

As is the case with any drug, gene delivery systems need to reach the affected tissues and cells to have a therapeutic effect. If this does not occur, there will be no therapeutic effect and may even result in iatrogenic complications, especially in the case of frequent re-administration of the delivery complex. To enhance therapeutic efficacy and reduce detrimental side effects, nucleic acid constructs must be efficiently and safely introduced into a specific cell populations, avoiding delivery into cells that are not therapeutic targets [12]. Consequently, one basic goal of non-viral gene therapy is the development of a cell-specific delivery system that delivers a therapeutic gene into selected cells [13].

The cell membrane and endocytic lysosomal shuttling system acts as a gatekeeper, selectively screening foreign materials entering the cell [14–16]. There have been extensive efforts to overcome the low cell penetration and intracellular delivery of genes. Previous studies have already shown that most of lipoplexes are internalized via the clathrin-dependent pathway [17] whereas the internalization of polyplexes can occur via the clathrin-dependent pathway, the caveolae-dependent pathway, (*i.e.*, macropinocytosis) or by both pathways simultaneously [18]. The endocytic pathway activated by a formulation will vary with the cell type, the molecular composition of the cell surface but also the physical properties of the delivery complexes [16,19]. A promising strategy to improve the intracellular efficacy is to enhance the uptake pathway specificity of non-viral vectors and this could be occurred by coupling chemically an appropriate ligand to the surface of the delivery formulation (e.g., transferrin receptors [20], asialoglycoprotein receptors [21]; mannose receptors [22], RGD peptides via integrins [23,24] …). The selection of the ligand-receptor pair is a key issue because the mechanism of intracellular transport and processing after endocytosis varies between cell types [25]. Consequently, the quality of synthesis and the characterization of the compound are some critical aspects to maximize its efficiency and its reproducibility.

Considering the folate receptor (FR), it is a glycosylphosphatidylinositol (GPI)-linked membrane glycoprotein with a molecular weight of 38–40 kDa [26,27]. Two membrane bound FR subtypes, FR-α (*Kd* ≈ 10^−10^ M) [28] and FR-β [29], have been identified in humans with a third FR subtype, FR-γ, that is secreted [29]. Despite its low expression in most non-cancerous human tissues [30], folic acid receptors offer many advantages: (1) high affinity and specificity [26,31]; (2) convenient availability and low cost; (3) a small targeting ligand, often leading to favorable pharmacokinetic properties of the folate conjugates and reduced probability of immunogenicity and (4) the receptor-ligand complex can be induced to internalize via potocytosis, a caveolin-coated endocytosis pathway [32,33]. Because folate-linked cargo’s of diameters <150 nm are efficiently bound and internalized by FR-expressing cells, it seemed reasonable to explore the possibility of using folic acid to facilitate liposomal vector delivery [34]. In the treatment of CF, one primary tissue target for gene transfer is the airway surface epithelium and the submucosal gland epithelium. Specifically, the ciliated cells at the luminal surface and the serous cells in the gland are believed to be the cellular targets that will best facilitate expression of the CF transmembrane conductance regulator (CFTR) protein [35]. Previously, folate moieties were conjugated to polyethylenimine (PEI) or the polyethylenglycol (PEG) chain of PEGylated PEI [36,37]. However, considering the high toxicity associated with PEI *in vitro* and *in vivo* [38,39], such delivery systems may not be therapeutically viable.

The present study describes the development of a family of non-viral cationic amphiphiles derived from glycine betaine [40] and the formulation of a series of neutral co-lipid analogues with a PEG tail and their functional characterization after the addition of a folate group [41]. Then, after having established the levels of FRs in various cell lines [HeLa, A549, 16HBE14o(−) and CFBE41o(−)], these formulations were tested *in vitro*. In particular, the targeting ability of the formulations was evaluated by comparing the transfection activity of lipids bearing a folate motif and lipids devoid of FA residue and, most importantly, by competitive inhibition assays by addition of free folate into the medium. In parallel, we also investigated the cytotoxicity impact of these lipoplexes. This study demonstrates that FA-modified DNA-cationic amphiphiles complexes are as efficient as classical cationic lipids, and no cytotoxicity was detected, unlike what was observed in the cationic controls. Additionally, local administration of the FA-complexes highlighted a effective transfection of the epithelial cells of the trachea as well as the nostril’s epithelium.

## 2. Results and Discussion

### 2.1. Lipids Synthesis and Lipoplex Preparation

**Figure 1:**
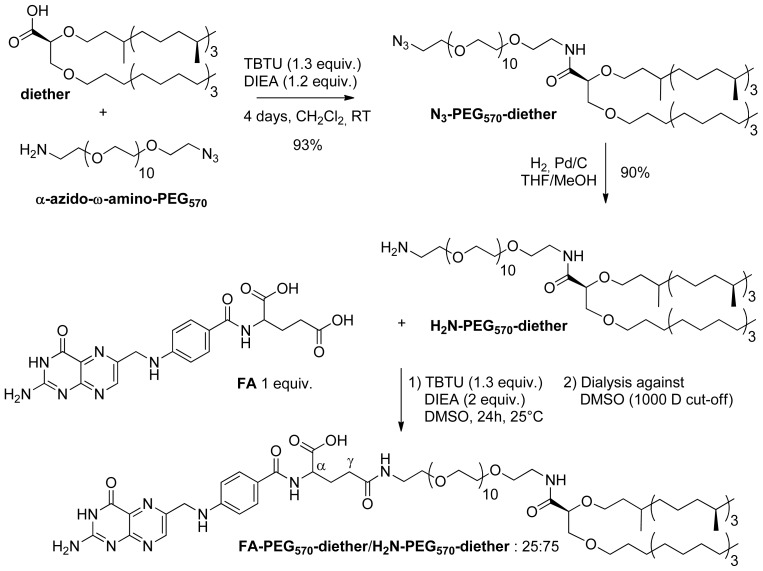
Synthesis of the H2N-PEG_570_-diether and FA-PEG_570_-diether.

The synthesis of the H_2_N-PEG_570_-diether and FA-PEG_570_-diether was achieved according to our preliminary studies [41,42]. The pegylated chain was introduced through a peptide coupling-type reaction between the carboxylic acid (diether) and the α-azido-ω-amino-PEG_570_ in the presence of TBTU and DIEA (Figure 1).

After four days of reaction, the N_3_-PEG_570_-diether was obtained in 93% yield. Pd/C-catalyzed hydrogenation of the azido function in THF/MeOH afforded the corresponding H_2_N-PEG_570_-diether in 90% yield. Then, our interest focused on the introduction of the FA into this pegylated diether. Indeed, we paid attention on the percentage of FA introduction and on the purification of the product, taking care of the total removal of unreacted folic acid. These precautions are essential for a targeting approach. Indeed, the number of ligands at the lipoplex surface plays a key role to promote the recognition and the internalization of the complexes [43]. Additionally, the presence of free folic acid into the formulation would compete with the FA-ligand and would result in an inhibition of the ligand-receptor interaction. This in mind, we carried out the introduction of the FA in the presence of 1 equivalent of H_2_N-PEG_570_-diether, 1.3 equivalent of TBTU and 2 equivalents of DIEA. After 24 h, the crude mixture was purified by dialysis against DMSO and was lyophilized. Thanks to NMR analysis, we demonstrated that the 1000 D cell-membrane cut-off permitted to remove totally the remaining free folic acid and afforded the corresponding FA-PEG_570_-diether in addition to H_2_N-PEG_570_-diether (25:75). Thus, we were able to have in hands perfectly defined samples of targeting lipids for which we knew the precise composition in folate ligand.

**Figure 2:**
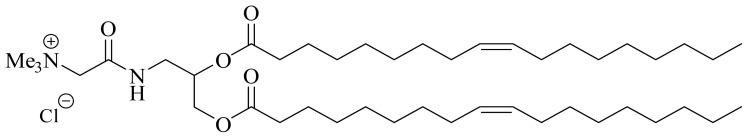
Structure of the cationic lipid MM18 derived from glycine betaine.

**Figure 3:**
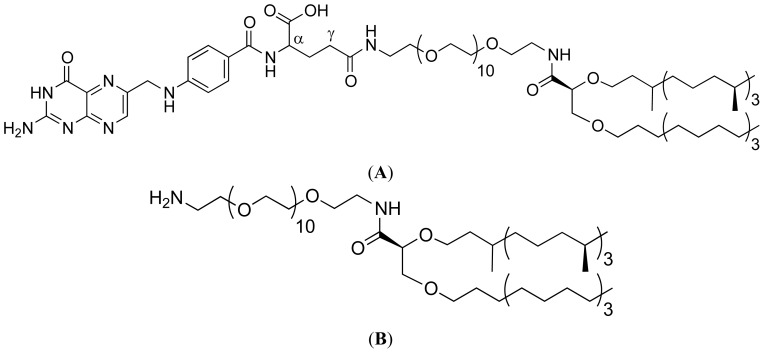
Diether PEG folate derivatives with (**A**, co-lipid **1**: FA -PEG_570_-diether) or without folate (**B**, co-lipid **2**: H_2_N-PEG_570_-diether).

Liposomes were then prepared by hydrating lipid films composed of the cationic lipid MM18 alone (liposomes) or combined with H_2_N-PEG_570_-diether or FA-PEG_570_-diether with water during 12 h at +4 °C followed by sonication (2 × 5 min) (Figures 2 and 3).

Lipid-DNA complexes were prepared by mixing the appropriate amounts of aqueous liposome suspensions with plasmid DNA expressing the luciferase reporter gene (pCMV-luc, 9.6 kb). Practically, to a fixed amount of DNA (4 μg), we added increasing amounts of cationic lipid MM18, MM18/H_2_N-PEG_570_-diether or MM18/FA-PEG_570_-diether formulations in order to form lipoplexes with increasing (+/−) charge ratios ranging from 0.5 to 8 (mean theoretical ratio of positive charges due to MM18 to negative charges of the DNA phosphate groups). The lipid-DNA complexes formed were analyzed after 30 min of incubation at room temperature.

### 2.2. Size and Charge Determination of the Liposomal Solutions and Lipoplexes

Before their use, the sizes and charges of the liposomes and the corresponding lipoplexes were precisely determined by dynamic light scattering (Table 1).

First, we measured that MM18 liposomes as well as formulations associating MM18 and H_2_N-PEG_570_-diether or FA-PEG_570_-diether formed some nanoparticles with a diameter between 100 and 170 nm, bearing a positive charge (between +39 to +54 mv). However, when pDNA was added to the liposomal solutions, sizes and charges of the lipoplexes were modified. Thus, whatever the co-lipid, the size was largely increased up to 500 to 600 nm, especially for the lowest charge ratio (*R* = 0.5). In addition, we observed that the proportion of co-lipid could decrease the size of the lipoplexes, influencing indirectly its transfection efficiency. Considering the charge of the complexes, we noticed that H_2_N-PEG_570_-diether lipoplexes were negative (−59 mv) for a charge ratio equals to 0.5. For the FA-PEG_570_-diether lipoplexes, whatever the percentage of co-lipid, they were negative for *R* = 0.5 (~(−60) mv) and *R* = 1 (~(−50) mv). At the neutral charge ratio, we assumed that the lipoplexes equipped with FA would mainly interact via the ligand-receptors way, whereas KLN47 and Lipofectamine, used as positive controls, should transfect mainly through non-specific electrostatic interactions due to the excess of positive charges. Such characterization of the lipoplexes, especially the formulations containing FA motifs, and not only liposomes, was of importance because it allowed to check the physical properties of the complexes and to appreciate the potential way of interaction, notably between the ligand and its receptors.

### 2.3. Expression and Localization of Folate Receptor α

**Figure 4:**
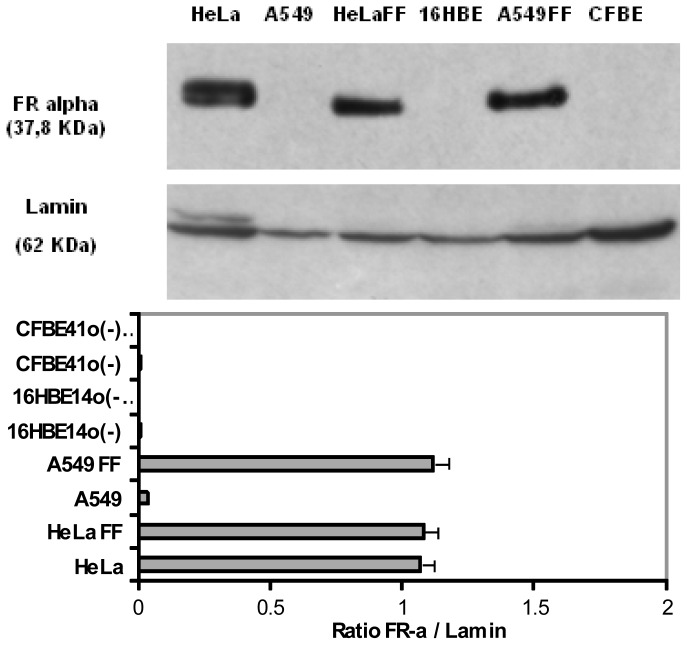
FR-α expression on HeLa, A549, 16HBE14o(−) and CFBE41o(−) human cells grown in standard medium or without folate into the medium. FF indicated Free-Folate medium. Lamin (62 kDa) was used as a deposit control. The FR-α expression level on HeLa was highly positive. On the opposite, the expression level of FR-α on A549, 16HBE14(o−) and CFBE41(o−) cell lines was very low or undetectable. The absence of folate into the medium induced a positive effect onto FR-α expression level in A549. Considering 16HBE14o(−) and CFBE41o(−) epithelial cells, none survived in such conditions. Using the software BIO-1D (Vilber-Lourmat, France), a quantification of the signal was realized and a ratio between the FR-α and lamin density was calculated.

**Figure 5:**
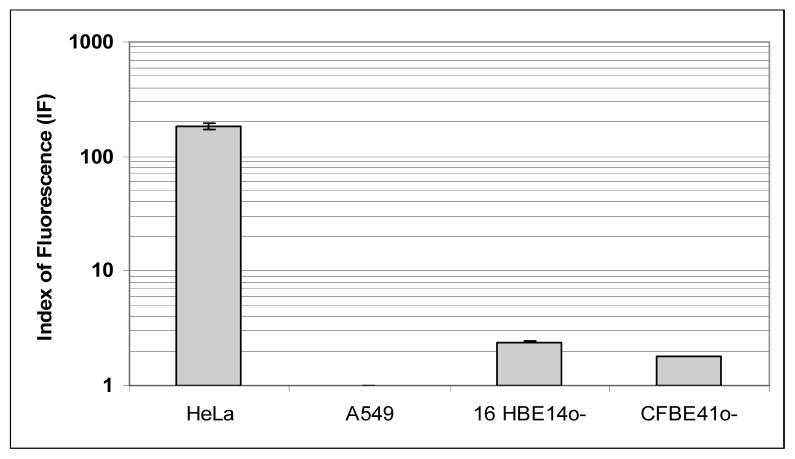
FR-α expression on HeLa, A549, 16HBE14o(−) and CFBE41o(−) cells evaluated by flow cytometry after indirect immunofluorescence labeling. Adapted from the protocol developed by Toffoli and co-workers., index of fluorescence was the ratio between medium immunofluorescence (IF) after anti FR-α labeling and isotype immunoglobulin labeling [44]. The FBP fluorescence index (FI) was defined as the mean of FBP-associated fluorescence divided by the isotopic control fluorescence. The FR-α expression was highly positive on HeLa (μ = 185.81; σ2 = 11.06), insignificant on A549 (μ = 1; σ2 = 0.01) and weakly positive on 16HBE14o(−) (μ = 2.42; σ2 = 0.03) and CFBE41o(−) (μ = 1.8; σ2 = 0.01).

**Figure 6:**
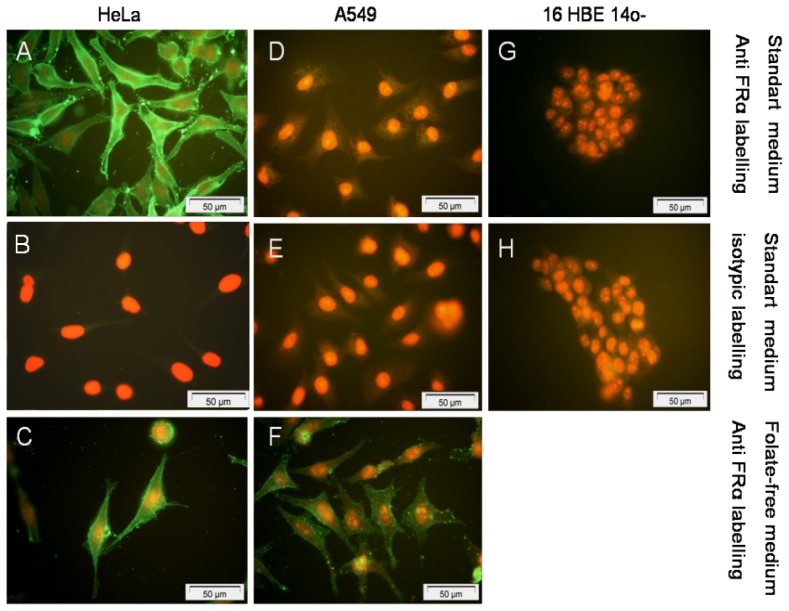
FR-α receptor expression on the membrane surface of HeLa, A549 and 16HBE14o(−) cells after indirect immunocytochemistry staining. Propidium iodide was used as nuclear counterstaining. The expression level of FR-α was high on HeLa with (**A**) or without (**C**) folate in medium *versus* control (**B**). In standard medium, FR-α receptor was poorly expressed in A549 (**D**) and 16HBE14o(−) (**G**) *versus* controls (respectively, **E** and **H**). Otherwise, when A549 were cultured in folate-free medium, FR-α expression was strongly increased (**F**). None picture was available for 16HBE14o(−) without folate because they did not support to be grown into folate free medium for more than 3 days.

To further interpret the transfection efficiency of the various formulations, we determined the level of FR-α expression and their localization onto a panel of human epithelial cells. First, whatever the techniques employed (Western blot, Flow cytometry assays and Immunofluorescence staining) (Figures 4, 5 and 6), we confirmed that HeLa cells strongly over-expressed FR-α.

**Figure 7:**
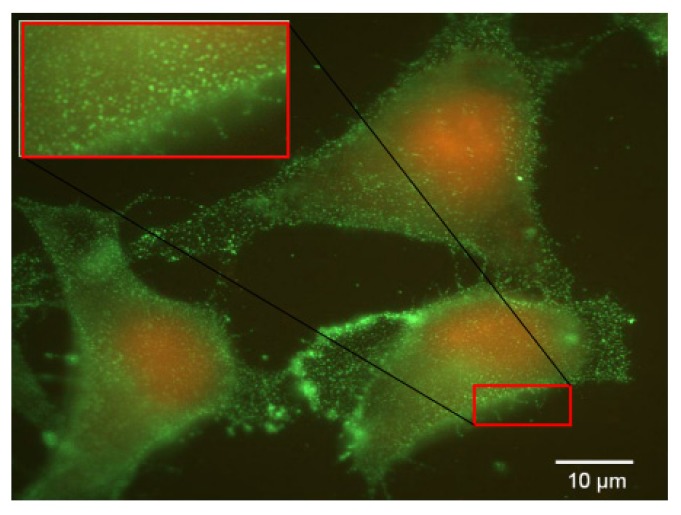
FR-α labeling by indirect immunocytochemical staining for A549 cells maintained in free folate medium. FR-α appeared as discrete clusters homogenously distributed at the cellular surface. This is more clearly illustrated in the left upper zoom (inset).

Considering the non-cancerous bronchial epithelial 16HBE14o(−) and CFBE41o(−) cells, they only expressed few FR-α receptors. This was shown trough Western blot assays as well as by indirect immuno-fluorescence analysis. Moreover, flow cytometry analyses confirmed that the expression was low. Considering A549 cells grown in a classical medium, none of these techniques allowed to report a significant expression. In order to establish if the FR expression could be promoted in respiratory epithelium, we submitted A549, 16HBE14o(−) and CFBE41o(−) cells to a very low concentration of folate in culture medium. In fact, the privation of folate into the culture medium for ten weeks made possible a FR-α over-expression in A549, at a level comparable to the one observed in HeLa cells (Figures 4 and 7). Considering 16HBE14o(−) and CFBE41o(−) cells, they did not survived in such conditions, probably due to their non cancerous status. Thus, the folate contained into the classical culture medium induced a low FR-α expression in A549 cell lines whereas these cells, maintained under low folate concentration, presented a FR-α distribution in the form of clusters at the cell surface (Figure 7).

Similar results were observed on HeLa cell line (Data not shown). Although such experimental settings were far from *in vivo* conditions, these observations were interesting to understand the FRα expression and the *in vitro* kinetic as well as its impact on transgene delivery.

### 2.4. Transfection Experiments and Targeting Properties

**Figure 8:**
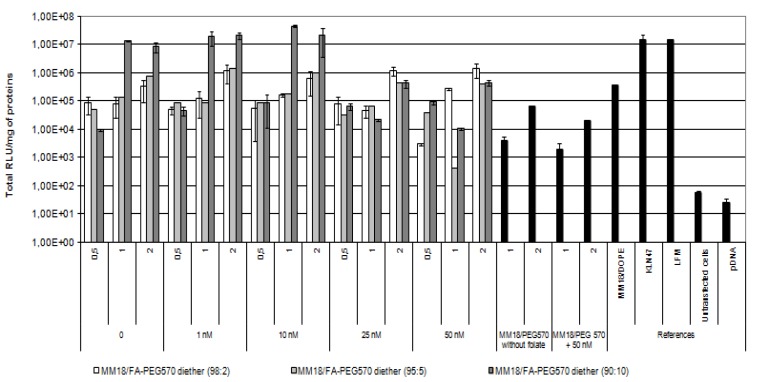
Evaluation of *in vitro* transfection efficiency of MM18/FA-PEG_570_ diether on Hela cell lines. Cells were transfected in 24-well plates as described in Materials and Methods. Naked DNA, KLN47, and LFM-based lipoplexes were used as controls and Luciferase activity was measured using a luminescent assay two days after transfection. Each data point indicates the mean value of Total RLU/mg of proteins obtained from three transfections and the standard deviation of this mean. To evaluate the strength of the ligand-receptor interaction, a growing folate concentration from 0 to 50 nM was added into the medium and luciferase activity was measured. We noted that the highest efficiency was obtained for *R* = 1 and beyond 10 nM, the transgene expression was strongly decreased.

As shown previously, there exists a large heterogeneity of FR-α expression between HeLa, A549 and 16HBE14o(−). Then, we submitted these epithelial cells to various complexes in order to evaluate their gene transfer ability. The transfection ability of the targeting complexes was first evaluated using HeLa cells, because of its high FR-α expression. The HeLa cells were transfected in 24-well plates as described in material and method, and the transgene expression was measured forty-eight hours after transfection. The specificity and affinity of the FA equipped complex was evaluated by using an increasing free folate concentration into the medium. The monocationic lipid KLN47, the commercial polycationic lipid (Lipofectamine), the formulations associating MM18 and H_2_N-PEG_570_-diether or DOPE were used as controls (Figure 8).

In such conditions, the highest transfection efficacy of the MM18/FA-PEG_570_-diether was observed for the highest proportion of co-lipid (90:10; *R* = 1). Its efficiency was quite similar to those observed with KLN47 and Lipofectamine. At R = 0.5, the transgene expression is lower than at *R* = 1, probably due to the lowest complexation strength of pDNA. We also observed a transgene expression when the complexes were employed at *R* = 2, highlighting the interest of lipoplexes measurements by dynamic light scattering. For the others proportions (98:2 and 95:5), their efficiency at *R* = 1 was respectively around 10^5^ and 10^6^ RLU/mg of proteins. However, beyond the addition of 10 nM free folate into the medium, the gene transfer capacity deeply decreased, down to 2 logs. Additionally, for the corresponding charge ratio, the transfection results of the MM18/H_2_N-PEG_570_-diether and MM18/DOPE complexes were less efficient than the FA-formulations. Consequently, the folate engraftment on a neutral co-lipid greatly increased the gene transfer ability of the cationic lipid, especially when added in a precise proportion.

**Figure 9:**
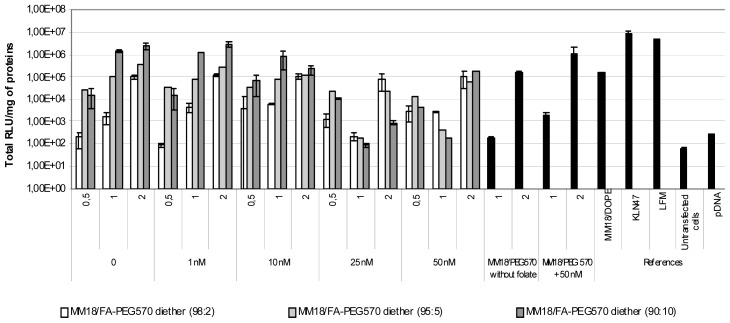
Transfection ability of FA-complexes on A549 cell line. A growing folate concentration from 0 to 50 nM in medium was tested. The efficiency decreased when the folate concentration was increased. The most efficient charge ratio was 1. MM18 cationic lipid formulated with H_2_N-PEG_570_-diether or with DOPE were less efficient. Targeting complexes were as efficient as KLN47 and Lipofectamine (LFM: ratio = 2).

On the A549 cell line, we obtained quite similar results (Figure 9), except that luciferase expression was about 1-log lower than on HeLa cells. In such condition, the highest transgene expression was also obtained with MM18/FA-PEG_570_-diether (90:10; *R* = 1). Despite of a low expression of the receptors, there exists an effective gene transfer, comparable to that obtained with Lipofectamine and KLN47. Beyond 10 nM of free folate added into the medium, the transfection efficiency was drastically decreased. This was probably due to the high receptor affinity for FA pattern that ensured the successful transfection of the MM18/FA-PEG_570_-diether complexes. Considering the MM18/H_2_N-PEG_570_-diether formulations, their transfection efficacy was increased with their charge ratio even in presence of folate into the medium.

**Figure 10:**
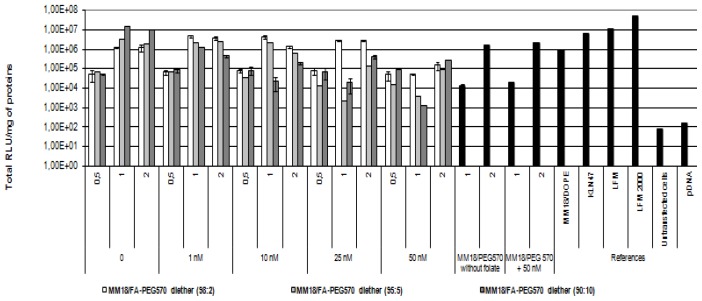
Transfection capacity of FA-complexes on 16HBE14o(−) cell line. The efficiency decreased as the folate concentration was increased. The most efficient charge ratio was 1 for the MM18/FA-PEG_570_-diether formulations. The same cationic lipid (MM18) with a colipid without folate (H_2_N-PEG_570_-diether) or with another colipide (DOPE) were less efficient at the same charge ration. KLN47 and Lipofectamine (LFM: ratio = 2) were as efficient as the targeting complexes.

Considering the 16HBE14o(−) cells, it is commonly accepted that they are difficult to transfect. Here, we reported that they were much more efficiently transfected by the targeting complexes than with cationic lipids chosen as positive controls (Figure 10).

The luminescence was as efficient as with FA-targeting complexes than using cationic lipoplexes such as KLN47 or Lipofectamine. Contrary to HeLa and A549, the highest transfection was reported for the lowest proportion of FA-PEG_570_-diether (*w:w*, 98:2) (~10^6^ RLU/mg of proteins) whereas the luciferase activity is around ~10^5^ RLU/mg of proteins for the 90:10 formulation. As a consequence, a higher concentration of free folate should be added to induce a decrease of the transgene expression, 50 nm being necessary to observe an effective decrease. In fact, for 95:5 and 90:10 formulations, a decrease was measured beyond 10 nM, as previously shown with the two other cell lines.

### 2.5. Relationship between Toxicity and Formulations

**Figure 11:**
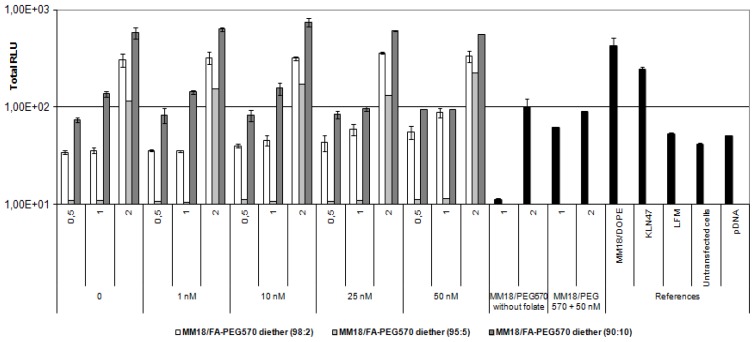
*In vitro* cytotoxicity of the FA-formulations on HeLa cells. Luminescent activity was measured 4 h after transfection. Each data point corresponds to the mean value of Total RLU derived from 3 transfections and the standard deviation of this mean. Cells mixed with naked uncomplexed DNA were used to evaluate the toxicity of pDNA. Untransfected cells were used to estimate the minimal cytotoxic level. We noted that MM18/FA-PEG570-diether was non-toxic at *R* = 1 but became toxic when used at *R* = 2, whatever the proportion of co-lipid.

**Figure 12:**
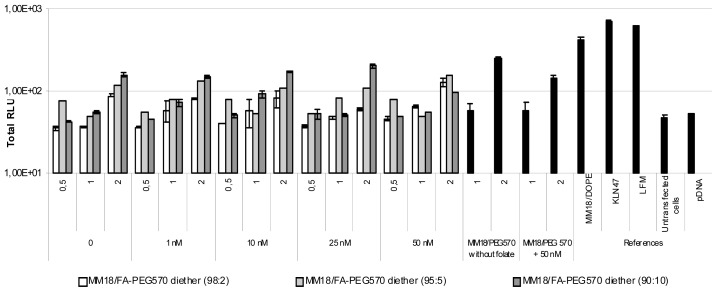
*In vitro* cytotoxicity of the FA-formulations on A549 cells. Earlier toxicity is correlated to the charge ratio. The FA-formulations have a lower toxicity than KLN47 and Lipofectamine.

**Figure 13:**
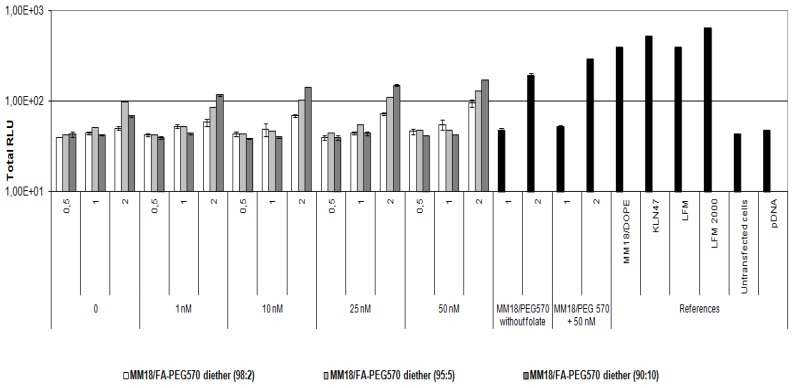
*In vitro* cytotoxicity of the FA-formulations on 16HBE14o(−) cells. The toxicity is especially increased with the positive charged complexes, notably the commercial references.

As shown in previous studies, the early cytotoxicity was directly correlated to the positive charge ratio of the complexes but was not associated with the various proportions of co-lipid. There was no toxicity for the neutral charges, whereas it was dramatically increased with the positively charged FA-complexes as well as with the cationic lipids used as references. Whatever the cell line tested, the toxicity of the targeting complexes at neutral of negative charge ratio was not different from untreated cells (Figures 11, 12 and 13).

### 2.6. Immuno-Localization of Folate Receptors on Mice’s Trachea and Nasal Cavities

**Figure 14:**
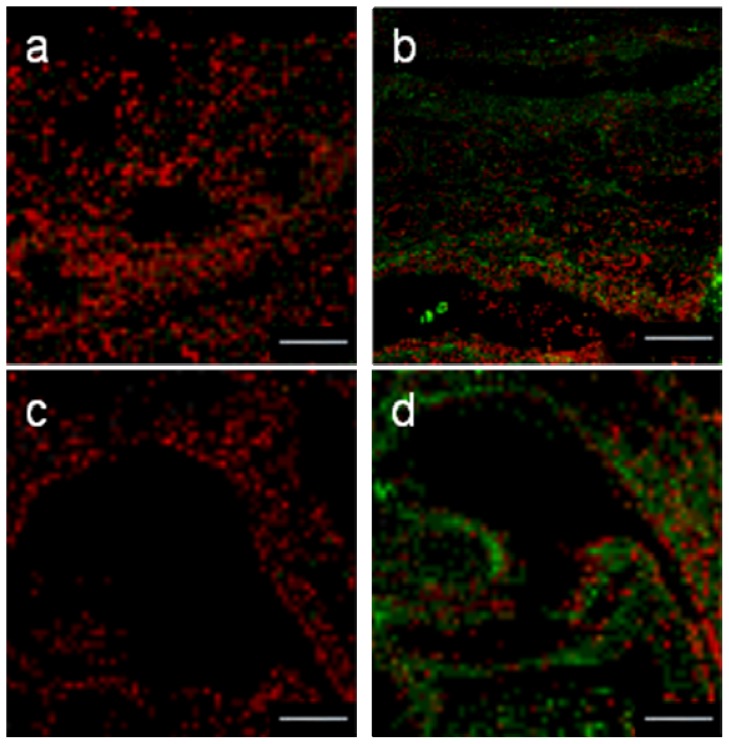
Comparison in the indirect immunodetection of FR-α. Cryocuts from nasal cavities (**a**,**b**) and mice trachea (**c**,**d**) were labeled or not with FR-α goat antibody (**b** and **d**). Red corresponds to Iodure propidium nuclei counter-staining. Bars = 200 μm.

To determine if such FA-derivated formulations could be used in vivo for a local administration, we performed some immunological assays to localize the FR-α on trachea and nasal cavities isolated from mice. As previously showed by Simmons *et al.* [45], we established here that the FR-α were well expressed in the upper airways. Then, they were located on the apical surface of the tracheal and nasal epithelial cells (Figure 14).

### 2.7. *In Vivo* Bioluminescence Imaging and Luciferase Activity in Trachea Homogenates

**Figure 15:**
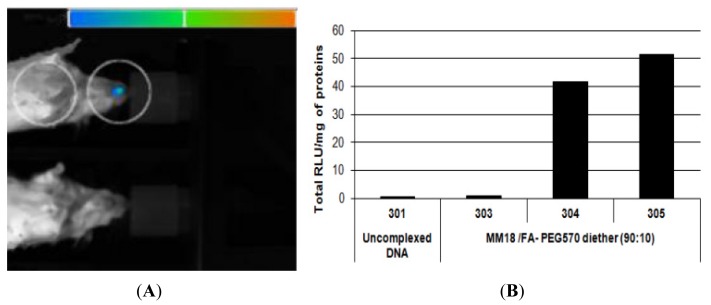
*In vivo* bioluminescence detection of luc expression 24 h after MM18/FA-PEG_570_ diether (90:10; *R* = 1) lipoplexes nasal administration (**A**). Luminescence images were superimposed onto still images of each mouse using the associated software (WinLight 32, Berthold, Stuttgart, Germany, 2007). (**B**) *In vivo* transfection efficiency of MM18/FA-PEG_570_ diether (90:10) based lipoplexes evaluated by luminescent measurements on trachea extracts Each mice received 50 μg of pDNA complexed or not. Results were expressed as RLU per mg of total proteins.

As FR-α were expressed at the apical surface of the epithelial cells, in vivo bioluminescence assays were performed to evaluate the transfection capacity of the MM18/FA-PEG_570_ diether (90:10; *R* = 1). As showed on Figure 15a, one mouse exhibited a positive bioluminescent signal in the nose area 24 h after a local nasal sniffing of the FA-formulation. Then, the luciferase activity was quantified on trachea homogenates 24 h after transfection. We measured that two mice presented a significant expression of the luciferase (Figure 15b) in comparison with mice treated with uncomplexed DNA. We concluded that this formulation was able to promote the transfection of cells into the nasal and tracheal epithelium. Finally, future studies should allow assessing more extensively the usefulness of gene delivery systems incorporating the FA-PEG_570_ diether co-lipid for efficient *in vivo* gene transfection, in particular with regard to the various routes of administration, the different cellular targets, and the general *in vivo* toxicity.

### 2.8. Discussion

Farther *in vitro* experiments, the possibility of producing nano-sized and stable lipoplexes that could be resistant to plasma protein interactions and capable of receptor-mediated targeting, internalization and subsequent transgene expression in specific cells is the subject of considerable investigations and would have major consequences for gene delivery. As non viral carriers are versatile, the fine tuning of their structure is of importance to improve their efficiency. Liposome-based delivery systems have long been identified as safe and effective drug carriers that are amenable to modification with cell-specific targeting ligands. In regards to gene delivery, the most common liposomes are those prepared with various cationic lipids in combination with a fusogenic lipid, such as DOPE.

After their sensitive synthesis and their physical characterization, one of the major limitations of folate-targeted liposomal gene therapy lies in the low rate of vector escape from intracellular compartments following folate receptor-mediated endocytosis. A folate-targeted, pH-sensitive, anionic liposomal vector was prepared to deliver DNA into folate receptor-bearing cells and discharged the pDNA into the cytoplasm, which preformed significantly more efficient than conventional pH-dependent liposomes [46]. Therefore, folate-linked targeting systems showed great potential for therapeutic applications [47]. Indeed, folate acid has been popularly used for intracellular delivering genes, imaging agents, and anti-cancer agents to folate receptor over-expressing tumour cells in a site specific manner.

For our investigation, we prepared FA-targeted co-lipid whom folate moiety was attached to the lipidic structure via PEG_570_ spacer. The PEG spacer was employed for two reasons. First, it was known that effective targeting of liposomes to cancer cells resulted when a 250 Å PEG spacer was used [48] Secondly, it was shown that having an additional negative charge closed to the FA enhanced the binding of corresponding conjugates to FR expressing cells [49].

After controlling the yield of the folate engraftment and performing a dialysis, we began our investigations. Among the physical parameters, the low poly-dispersity revealed a quite homogenous nano-population. We also controlled that lipoplexes for the low charge ratio were effectively negatively charged. As our objectives were to demonstrate the efficiency and the specificity of folate targeting on healthy bronchial cell lines as well as in vivo, we first checked the FR-α expression on the various cells, cancerous or not. As previously shown in the literature, we observed a high expression of FR-α in HeLa cells, whereas the expression was quite low in A549 and 16HBE14o(−). Low folate expression on the non-cancerous cell lines as 16HBE14o(−), did not enhance research for folate targeting on theses cells. However, most of them have at least few folate receptors. New targeting therapy as cetuximab (anti-EGFR) demonstrates that more than membrane receptor expression, intracellular mechanisms are the main limiting factors for therapy efficiency.

Here, we reported that Folate-equipped DNA complexes mediated efficient gene transfer into human epithelial cells with a necessary adaptation of the formulation depending on cell type. In term of efficiency, these FA-complexes led to a luciferase expression comparable to the cationic references as KLN47 and Lipofectamine. In addition, no cytotoxicity was detected, unlike what was observed in the cationic controls, probably due to their neutral charge ratio. It has previously been shown that co-incubation of folate-targeted liposomes with large amounts (~50 nM) of folic acid was required to significantly reduce FR-specific cellular liposome uptake, suggesting that folate liposomes may have an unusually high affinity for FR-positive cells [50]. In this report, we demonstrated that this effect was observed for lower amounts of folic acid (beyond 10 nM) depending on the formulation and the FR-α wealth. In front of these encouraging results, we looked first at the presence of FR-α in the trachea and the nostril of mice. As previously observed [45], we established that FR-α were well expressed in these tissues and located at the apical surface, opening the way to local administration assays. Here, we reported that the MM18/FA-PEG_570_ diether (90:10) was able to promote an effective expression of the reporter gene both by bioluminescent imaging and luciferase measurements. Thus, such formulations could be of interest in CF gene therapy. In larger ongoing experiments, we are at present investigating the airway cell type(s) actually transfected with the FA-based lipoplexes.

In past few years, receptor mediated intracellular drug delivery received major attention in modern gene delivery therapeutics, although the approach was just at nascent stage, it showed a promise to develop DNA-based pharmaceuticals for the effective managements of gene associated disorders in near future.

## 3. Experimental Section

### 3.1. Cell Culture

For these experiments, various human epithelial cell lines were used (Hela, A549, 16HBE14o(−) and CFBE41o(−)). Both cancerous cell lines (HeLa, n° ccl 2 and A549, n° ccl 185) were obtained from the American-Type Culture Collection (Rockville, MD, USA). Cells were maintained in D-MEM (Gibco-BRL, Cergy Pontoise, France) supplemented with 10% fetal calf serum (FCS) (Cambrex, Verviers, Belgium), 2 mM glutamine, 100 U/mL of penicillin, 100 U/mL of streptomycin, and 1% fungizone. Considering the non cancerous human bronchial epithelial cell lines (16HBE14o(−) and CFBE41o(−)), they were kindly provided by Dr. D. Gruenert (University of San Francisco, San Francisco, CA, USA). These cells were seeded on coated flask with a fibronectin, collagen and BSA solution and grown in E-MEM (Gibco-BRL, Cergy Pontoise, France) supplemented with 10% FCS, 2 mM glutamine, 100 U/mL of penicillin, 100 U/mL of streptomycin, and 1% fungizone. All cells were maintained in 5% CO_2_ and at 37 °C between 10^5^/mL and 10^6^/mL. Concerning the FR promotion assays, all the cells were maintained into Folate Free Medium RPMI 1640 (FDRPMI).

### 3.2. Plasmid

Plasmid pTG11033 (a gift from Transgene, Strasbourg, France) containing the luciferase gene, under the control of the cytomegalovirus immediate early gene 1 (IE1-CMV) promoter, intron 1 of the HMGCoAR (Hydroxymethyl-glutaryl-CoA reductase) gene and the simian virus 40 (SV40) poly(A) signal was used. It was amplified in the DH5α strain of *E. coli* and then isolated by alkaline lysis and purified with the Qiagen Endo Free Giga Kit (Qiagen, Courtaboeuf, France) according to the manufacturer’s protocol. It was dissolved in endotoxin-free water and stored at −20 °C. Plasmid DNA was quantified by spectrophotometry at an optical density of 260 nm and was confirmed to be free of protein contamination, with A260/280 ratio between 1.8 and 2.

### 3.3. Cationic Lipids, Folate Equipped Co-Lipids: Synthesis and Formulations

Glycine betaine-based cationic lipid MM18 (Figure 2) was previously used as an efficient monocationic lipid for gene transfer [40]. Considering the folate engraftment, it was wrongly considered by many authors as relatively simple and most of them did not precise that the purification step was difficult and did not indicate the yield of their reactions. After optimizing the different steps of the synthesis, a satisfying yield was finally obtained (~90%) and was followed by a dialysis purification to remove all the free folate [41]. Two original pegylated diether co-lipids inspired from archaelipids were herein synthesized (H_2_N-PEG_570_-diether and FA-PEG_570_-diether), whom one equipped with a FA pattern (Figure 3) and then, formulated with MM18 in various proportions (*w:w*; 98:2; 95:5 and 90:10).

The length of the PEG chain (10 ethylene oxide units) was chosen as a balance (1) to facilitate the FA–FR interaction without any steric hindrance due to the lipoplex and (2) to allow an easy preparation of the liposomes despite the presence of PEG chains [51]. Size measurements by using dynamic light scattering were performed on both liposomes and lipoplexes’ formulations to control the negativity of the complexes at low charge ratio.

Liposomes were then prepared by hydrating lipid films composed of bilayer-forming cationic lipid MM18 alone (liposomes), combined with a co-lipid possessing or not a FA pattern or with the referent co-lipid DOPE, with water for 12 h at 48 °C followed by sonication (2 × 5 min). Then, the formulations were stored at +4 °C. Before use, small unilamellar vesicles (SUVs) of each compound were prepared by sonication for 10 min in a sonicator bath (Prolabo, Paris, France) [52]. To prepare the lipoplexes, plasmid DNA was first diluted in sterile pyrogen-free DI water and added to the lipid solution. All the lipoplexes were kept at room temperature at least 30 min before being used for *in vitro* assays.

KLN47 (Platform IBiSA «SynNanoVect», Brest/Rennes, France) [53,54] and Lipofectamine reagent (indicated as LFM) (Invitrogen, Cergy-Pontoise, France) were used as positive controls at a charge ratio of 4 for KLN47 and 2 for (LFM), whereas uncomplexed pDNA and untreated cells were used as negative controls.

### 3.4. Determination of the Charge Ratio of the Lipoplex

The charge ratio (R) was calculated theoretically as the molar ratio of lipid formulations (one positive charge per molecule) to phosphate nucleotide residues (average MW ~330). In this study, various charge ratios (+/−) were prepared from 0.5 to 8 with a constant amount of pDNA delivered (4 μg). The formulations were then evaluated for their transfection efficiency and cellular toxicity depending on the cell type. As mentioned above, the sizes and the charges of the liposomes and lipoplexes were determined by dynamic light scattering (Malvern instruments, Orsay, France).

### 3.5. *In Vitro* Transfection Protocol

The transfection activity of the lipoplexes *in vitro* was assessed using various cell lines. Cells were seeded onto a 24-well tissue culture plate at 100,000 cells per well (three wells per condition tested) (Corning Costar, Cambridge, MA, USA). To limit the interactions of the folate contained into the medium and the formulations, cells were seeded for 24 h before transfection in folate deficient-RPMI medium 1640 (FDRPMI 1640) (Life Technologies, Carlsbad, CA, USA) supplemented with 10% heat inactivated fetal bovine serum (HIFBS) and incubated overnight in a humidified 5% CO_2_ atmosphere at 37 °C. Transfection of the cells was performed as described by Felgner *et al.* with the following modifications [50]. Thus, 4 μg of pDNA were mixed with the appropriate amounts of liposomes in 700 μL of FDRPMI. The tube was incubated for 30 min at room temperature and the complex was then added to each well. All the controls were also prepared in FDRPMI. After four hours at 37 °C, the medium was removed and replaced by fresh medium containing 10% HIFBS. The cells were then re-incubated at 37 °C for 48 h prior to luciferase detection and cytotoxicity measurements. Considering inhibition experiments, four concentrations of folate free (Sigma, St. Louis, MO, USA) were herein tested: 1 nM, 10 nM, 25 nM and 50 nM (in final concentration). The addition of folate into the medium was performed just before the addition of the lipoplexes into the culture dish.

### 3.6. Luminescent Detection of Luciferase

Forty-eight hours after transfection, the cells were assayed for luciferase expression using a chemiluminescent assay (Promega, Charbonnière, France). Tests were performed as described by the manufacturer. After one wash in PBS1X, cells were treated with 200 μL Lysis Buffer (Promega) for 30 min. The supernatant was then distributed into the wells of a 96-well opaque plate. Luciferase activity in the supernatant was quantified by a luminometer (MLX Microtiter Plate Luminometer, Dynex, Guyancourt, France), measuring light emission over a 15-s reaction period. The results were expressed in total relative light units (Total RLU) per mg of protein (Total RLU/mg of total protein). The total protein concentration of each supernatant was measured using a BC Assay protein quantification kit (Interchim, Montluçon, France). The positive control values were generated by transfection of the cells with Lipofectamine and KLN47 and the negative control values were established by transfection of cells with pDNA alone. We also measured the basal value of the untreated cells. Quantitative variables were expressed as mean ± standard error of the mean (S.E.). Differences in the means between the groups were assessed by using the Student’s *t* test. A *p* value of less than 0.05 was considered as significant.

### 3.7. Determination of Cell Toxicity

The early toxicity of the different formulations was determined by the chemiluminescent ToxiLight cytotoxicity assay (Cambrex, Emerainville, France) as described below. ATP detection systems were commonly used for detecting cytotoxicity. These measured the ATP from viable cells (or lack of them) rather than a direct measurement of cytotoxicity, and in some cases, cell death may not be the correct assumption to make when ATP levels decrease. An alternative indicator of cytotoxicity was damage to the plasma membrane. This allowed reagents to enter the cells and allows leakage of cell components into the surrounding medium. One of the enzymes released when cell membrane damage occurs was adenylate kinase (AK), a ubiquitous protein present in all eukaryotic cells. The ToxiLight Assay was based on the bioluminescent measurement of adenylate kinase. The measurement of the release of AK from cells allowed the accurate and sensitive determination of cytotoxicity and cytolysis.

Thus, four hours after transfection, 20 μL of supernatant was taken from each well and was then distributed into the wells of a 96-well opaque plate. One hundred microlitres of AK Detection Reagent (reconstituted in Tris-Ac Buffer) was then added to each well and incubated at room temperature for 5 min. The results were expressed in relative light units and untransfected cells were used as a reference.

### 3.8. Western Blot Assays

To determine the level of α-folate receptor (FR-α) expression in the different cell lines, western blot assays were performed. Cells were removed from flask by trysinisation and washed twice with PBS1X. Then, the lysis buffer (10 mM Tris, 150 mM NaCl, 2 mM EDTA, 0.2% SDS, 1 mM PMSF, and Protease Inhibitor Cocktail, Sigma-Aldrich) was added. The lysates were regularly vortexed and maintained at +4 °C, and then centrifugated at 10,000× *g* for 20 min at +4 °C. The protein content was measured by the Bradford method (BC Assay protein quantification kit) (Interchim, Montluçon, France) and the samples were stored at −20 °C until required. In each well of the SDS-PAGE gel, 30 μg of total protein were loaded. The samples were separated electrophoretically by 10% SDS-PAGE. After electrophoresis, the proteins were transferred to the membrane (Hybond P, GE Healthcare, Velizy, France) by applying 150 V for ninety minutes. The membrane was incubated with 3% BSA–0.1% Tween–PBS1X overnight at +4 °C. The membrane was first incubated for one hour with monoclonal antibody against FRα (MAb MOv 18, ALX-804-439) (Enzo Life Sciences, Villeurbanne, France) (dilution = 1:8000 in 3% BSA–0.1% Tween–PBS1X) and then it was immersed in a solution containing the secondary antibody (Polyclonal antibody to Mouse IgG1-HRP, ALX-211-203) (Enzo Life Sciences, Villeurbanne, France) (dilution = 1:20000). Membrane was revealed with a chemiluminescence detection system (kit ECL + western blotting detection system) (GE Healthcare, Velizy, France). Considering the controls, after three washes with 0.1% Tween–PBS1X, the membrane was incubated for one hour with monoclonal antibody against Lamin (sc-6215) (Santa Cruz Biotechnology, San Diego, CA, USA) (dilution = 1:1000 in 3% BSA–0.1% Tween–PBS1X) and then it was immersed in a solution containing the secondary antibody (Donkey polyclonal to Goat IgG-H&L (HRP), ab7125) (Abcam, Paris, France) (dilution = 1:20000). Membrane was revealed with a chemiluminescence detection system (kit ECL and western blotting detection system) (GE Healthcare, Velizy, France).

### 3.9. Flow Cytometry Assays

To precise the expression of FR-α in the human epithelial cells, we followed and adapted the protocol from Toffoli and co-workers [44] based on flow cytometry assays. First, cells were gently removed from plates by trypsination. Cells were then incubated with the MAb MOv 18 (human IgG1) (dilution = 1:20) or the isotopic control mouse (Purified mouse IgG1-k isotype control BD pharmingen) (dilution = 1:10) (Becton Dickinson, city, France) in a total volume of 50 μL for 1 h at 4 °C. The primary antibody was eliminated by centrifugation and the FITC-conjugated sheep anti-mouse IgG1 (Sigma, Milan, Italy) was added for 15 min at a dilution of 1:12. After a final wash, samples were re-suspended in 500 μL of PBS1X and analyzed by a FACScan flow cytometer (FACScan, CellQuestPro, Becton-Dickinson, Le pont de Claix, France). For each measurement, 10,000 events were collected. The FBP fluorescence index (FI) was defined as the mean of FBP-associated fluorescence divided by the isotopic control fluorescence.

### 3.10. Immuno-Localization of Folate Receptors on Human Epithelial Cells

In order to localize the folate receptors into the cellular structure, cells were grown on poly-L-lysine coated glass slides for 48 hours. Then, they were fixed by a 3.7% paraformaldehyde solution for 10 min. After washing in PBS1X, a double immunocytochemical stained was performed (primary antibody: MAb MOv 18 IgG1, dilution = 1:100 or isotopic control mouse, dilution = 1:100; secondary antibody: FITC-conjugated mouse anti-human, dilution = 1:100 or FITC-conjugated sheep anti-mouse, dilution = 1:100). Propidium iodide was used as nuclear counterstaining.

The slides were observed by fluorescent microscopy (Olympus, Rungis, France). Negative controls included the omission of the primary antibody and showed no staining. To study the impact of the medium on FR-α expression, we also performed the immunostaining of cells grown in folate-free medium (FDRPMI).

### 3.11. Immunohistological Assays from Mice’s Trachea and Nasal Cavities

After sacrificing six week-old Swiss mice, their trachea and nasal cavities were quickly infused with 1X PBS and were then immersed in isopentane at −180 °C. Sections of 5μm-tick were realized and fixed in 4% formaldehyde. Unspecific sites were saturated for 1h with donkey serum (Ref UP 77719A, Interchim, Montluçon, France) (1:10 in PBS1X). Slides were then incubated with FR-α goat antibody (Ref sc-16389, Santa Cruz biotechnology) diluted at 10% in PBS1X for 24 h at 4 °C. They were then washed four times with PBS. For the negative controls, the primary antibody was not dropped onto the sections. All the slides were incubated with the donkey anti-goat secondary antibody (Ref FP-DAGOTTGX 488 Interchim, Montluçon, France) (1:20 in PBS1X) for 45 min and then washed three times in PBS1X under gentle agitation. Propidium iodide was used as nuclear counterstaining.

The slides were observed by fluorescent microscopy (Olympus, Rungis, France).

### 3.12. Bioluminescence Imaging and Luciferase Activity in the Trachea Homogenates

In addition, concerning the nasal airway epithelium which is difficult to harvest, we administered locally FA-lipoplexes containing the luc-expressing pTG11033 plasmid in order to confirm transgene expression by *in vivo* bioluminescence imaging. Thus, as regards nasal administration, each mouse received 50 μg of luc-expressing plasmid complexed with MM18/FA-PEG_570_ diether (90:10; *R* = 1) in a final water volume of 50 μL. Six to nine weeks old female Swiss mice (Janvier breeding center, Le Genest Saint Isle, France) were housed and maintained at the University animal facility; they were processed in accordance with the Laboratory Animal Care Guidelines (NIH publication #85-23 revised 1985) and with the agreement of the regional veterinary services (authorization FR; 29-024). The mice were anesthetized with a 2% air-isofluran blend through a nose cone and the complexes were dropped off the nostrils.

Mice to be imaged received first an intraperitoneal injection of luciferin (4 mg in 200 mL Hepes Buffer (20 mM); Interchim, France). Three minutes later, the animals were anesthetized with a 4% air-isofluran blend. Once laid in the acquisition chamber, the mice were maintained anesthetized with a 2% air-isofluran mixture all along the experiment. Five minutes after luciferin injection, luminescence images were acquired using an in vivo imaging system (NightOWL NC320, Berthold) and associated software (WinLight 32, Berthold) with a binning of 8 × 8 and an exposure time of 4 min. Luminescence images were then superimposed onto still images of each mouse.

Twenty four hours after transfection, the mice were killed by cervical dislocation and their lungs were removed for analysis. Luciferase expression was evaluated as previously described. Complete lysis was achieved by vigorous shaking at 4 °C for 45 min and the supernatant was isolated by centrifugation. Luciferase activity and total protein content were then evaluated as indicated before. Results were expressed as RLU per mg of total proteins.

## 4. Conclusions

This work was meant to be an extension of encouraging results described in the literature that showed that grafting of folic acid residues to DNA/lipid (or polymers) complexes led to selective expression of foreign genes in folate-expressing cells *in vitro* [49,55]. After formulating and characterizing the folate-modified DNA nanocomplexes, we showed that such formulations led to luciferase transgene expression levels comparable to cationic lipids. Moreover, no cytotoxicity was detected with these neutral complexes, in comparison with cationic complexes. The addition of free folate leading to the decrease of the luciferase expression around 50%, this transfection mechanism appeared to be partially due to endocytosis mediated to folate receptor. To the best of our knowledge, these results have had no in vivo success so far. As the size and formulation of DNA complexes being a key factor for successful delivery *in vivo*, we focused on DNA condensation to this goal. This approach seems to be promising as, after having confirmed the expression of FRα-along the upper airways, we performed some preliminary intranasal instillation and few mice were found positive as assessed using *in vivo* bioluminescence imaging techniques. Additional *in vivo* experiments are ongoing to confirm the potential of such FA-formulations.

## Figures and Tables

**Table 1:** Sizes and charges of the liposomal formulations and lipoplexes.

	MM18	H_2_N-PEG_570_-diether (98:2)	H_2_N-PEG_570_-diether (95:5)	H_2_N-PEG_570_-diether (90:10)	FA-PEG_570_-diether (98:2)		FA-PEG_570_-diether (95:5)		FA-PEG_570_-diether (90:10)
Average diameter (nm)	106	93	148	167	92		150		170
Polydispersity index	0.211	0.273	0.275	0.245	0.255		0.261		0.112
Zeta potential (mV)	+54	+39	+42	+44	+45		+49		+45

	**MM18 + DNA (4 μg)** ***R*= 2**	**H_2_N-PEG_570_-diether + DNA (4 μg) (95:5)** ***R*= 0.5**		**H_2_N-PEG_570_-diether + DNA (4 μg) (95:5)** ***R*= 1**	**H_2_N-PEG_570_-diether + DNA (4 μg) (95:5)** ***R*= 2**		**H_2_N-PEG_570_-diether + DNA (4 μg) (95:5)** ***R*= 4**		**H_2_N-PEG_570_-diether + DNA (4 μg) (95:5)** ***R*= 8**

Average diameter (nm)	571	685		524	570		510		573
Polydispersity index	0.211	0.281		0.205	0.235		0.209		0.248
Zeta potential (mV)	+41	−59		+39	+34		+37		+39

	**FAPEG_570_-diether + DNA (4 μg) (98:2)** ***R*= 0.5**	**FAPEG_570_-diether + DNA (4 μg) (98:2)** ***R*= 1**	**FAPEG_570_-diether + DNA (4 μg) (98:2)** ***R*= 2**	**FAPEG_570_-diether + DNA (4 μg) (95:5)** ***R*= 0.5**	**FAPEG_570_-diether + DNA (4 μg) (95:5)** ***R*= 1**	**FAPEG_570_-diether + DNA (4 μg) (95:5)** ***R*= 2**	**FAPEG_570_-diether + DNA (4 μg) (90:10)** ***R*= 0.5**	**FAPEG_570_-diether + DNA (4 μg) (90:10)** ***R*= 1**	**FAPEG_570_-diether + DNA (4 μg) (90:10)** ***R*= 2**

Average diameter (nm)	865	662	677	244	302	175	427	521	163
Polydispersity index	0.333	0.266	0.240	0.232	0.225	0.214	0.190	0.468	0.215
Zeta potential (mV)	−56	−36	+38	−59	−34	+43	−53	−49	+47
